# Contrasting genetic structure of rear edge and continuous range populations of a parasitic butterfly infected by *Wolbachia*

**DOI:** 10.1186/1471-2148-13-14

**Published:** 2013-01-18

**Authors:** Dario Patricelli, Marcin Sielezniew, Donata Ponikwicka-Tyszko, Mirosław Ratkiewicz, Simona Bonelli, Francesca Barbero, Magdalena Witek, Magdalena M Buś, Robert Rutkowski, Emilio Balletto

**Affiliations:** 1 Department of Life Sciences And Systems Biology, University of Turin, Via Accademia Albertina 13, Torino, I-10123, Italy; 2 Department of Invertebrate Zoology, Institute of Biology, University of Bialystok, Świerkowa 20B, Białystok, PL-15-950, Poland; 3 Department of Human Reproduction Biology and Pathology, Institute of Animal Reproduction and Food Research, Polish Academy of Sciences, Skłodowskiej-Curie 24a, Białystok, PL-15-276, Poland; 4 Department of Vertebrate Zoology, Institute of Biology, University of Bialystok, Świerkowa 20B, Bialystok, PL-15-950, Poland; 5 Department of Molecular and Biometrical Techniques, Museum and Institute of Zoology, Polish Academy of Sciences, Wilcza 64, Warszawa, PL-00-679, Poland

**Keywords:** *Maculinea arion*, *Wolbachia*, Rear edge, COI, EF-1α

## Abstract

**Background:**

Climatic oscillations are among the long-term factors shaping the molecular features of animals and plants and it is generally supposed that the rear edges (i.e., the low-latitude limits of distribution of any given specialised species) situated closer to glacial refugia are vital long-term stores of genetic diversity. In the present study, we compared the genetic structure of several populations of an endangered and obligate myrmecophilous butterfly (*Maculinea arion*) from two distinct and geographically distant parts of its European distribution (i.e., Italy and Poland), which fully represent the ecological and morphological variation occurring across the continent.

**Results:**

We sequenced the COI mitochondrial DNA gene (the ‘barcoding gene’) and the EF-1α nuclear gene and found substantial genetic differentiation among *M. arion* Italian populations in both markers. Eleven mtDNA haplotypes were present in Italy. In contrast, almost no mtDNA polymorphisms was found in the Polish *M. arion* populations, where genetic differentiation at the nuclear gene was low to moderate. Interestingly, the within-population diversity levels in the EF-1α gene observed in Italy and in Poland were comparable. The genetic data did not support any subspecies divisions or any ecological specialisations. All of the populations studied were infected with a single strain of *Wolbachia* and our screening suggested 100% prevalence of the bacterium.

**Conclusions:**

Differences in the genetic structure of *M. arion* observed in Italy and in Poland may be explained by the rear edge theory. Although we were not able to pinpoint any specific evolutionarily significant units, we suggest that the Italian peninsula should be considered as a region of special conservation concern and one that is important for maintaining the genetic diversity of *M. arion* in Europe. The observed pattern of mtDNA differentiation among the populations could not be explained by an endosymbiotic infection.

## Background

The currently observable molecular biogeography of animals and plants has been historically shaped by the evolutionary history of individual species and recently by their distinctive demographic features. The most important long-term factors that influence European molecular biogeography are related to climatic oscillations [1]. The Pleistocene glacial and interglacial cycles caused extensive latitudinal and altitudinal shifts in species distribution ranges because of the species’ need to track shifts in their habitats. Therefore, during the ice ages many species that are currently widely distributed in Europe were confined to the southern extremities of the three major Mediterranean peninsulas, i.e., Iberia, Italy and the Balkans [2]. Repetitive range contractions and expansions affected the genetic structure of these species, but the real impact of such processes varied widely based on the habitat requirements and dispersal abilities of each species [3,5]. Mobile and ubiquitous species typically show relatively homogenous genetic structures compared with sedentary and specialised species. Representatives of the latter group usually disperse in a stepping-stone manner and are frequently more affected by stochastic processes; they are most likely to experience losses of neutral genetic variation and changes in allele frequencies. Therefore, for sedentary and/or specialized species, higher genetic diversity is often observed in areas situated closer to glacial refugia than at the northern edges of a species’ range [6] and references therein.

Hampe and Petit [7] suggest that rear edges, which are defined as the low-latitude limits of a species’ distribution, are vital long-term stores of the genetic diversity of species. The rear edge populations are usually small in size and are thus characterised by low genetic diversity and high inter-population genetic differentiation (e.g. the rear-edge hypothesis) [8]. According to Bilton et al. [9], such relic populations have preserved their genetic distinctiveness but have not been the source of major postglacial re-colonisations. Furthermore, the rear edges are relatively stable due to heterogeneous topography, which allowed the species to find suitable climatic conditions with local elevation shifts [10]. Such a scenario is suspected to have occurred in northern Italy for the highly specialised butterfly *Maculinea arion* (Linnaeus, 1758), which was potentially able to survive glaciations at the base of the Alps and, at the end of the Ice Age, was able to either re-colonise the higher altitudes following shifts of its habitat or adapt to the new biotopes in the lowlands [11].

*M. arion* lives as an obligatorily myrmecophilous species, and its survival depends on the concurrent presence of two types of resources: specific food plants and specific host ants. Its caterpillars, after spending a short period (10-15 days) feeding on *Thymus* spp. or *Origanum vulgare*, leave the plant and reach the ground at the beginning of their fourth (final) instar. There, larval survival is limited by required discovery by a forager ant of the genus *Myrmica*, which adopts it and takes it to its nest where the larva will spend winter and complete its development by feeding on the ant’s brood [12]. Socially parasitic relationships with ants can be specific to the local species [13,14] and are promoted by chemical and acoustical mimicry [15,16].

Its complex life cycle makes *M. arion* sensitive to subtle environmental changes; therefore, this species is now threatened in many countries. *M. arion* became extinct in Britain, and it was later successfully reintroduced after full habitat restoration [13]. On a European scale, the status of *M. arion* has worsened during the last decade, from near threatened [17] to endangered [18]. *M. arion* is listed in Annex IV of the Habitat Directive and is considered an important indicator of habitat quality and stability as well as an umbrella species for certain types of grassland communities [19,21] as its protection assures an indirect benefit to many other species. Despite its specialised life history and conservational status, this lycaenid shows considerable variation in morphology and ecology across its range, which encompasses vast areas of the Palearctic from Central Spain to Japan [22]. In Europe, *M. arion* inhabits various types of warm and dry grasslands at elevations between 50 and 2500 m [21,23,25].

In the present research, we analysed and compared the genetic differentiation of twenty *M. arion* populations from two distinct and distant parts of this species’ European distribution (Italy and Poland), which fully represent the ecological and morphological variation of the butterfly across the continent. The Polish populations, which inhabit xerothermic meadows, occur on southerly exposed slopes or sandy flat areas, and exploit *Thymus* spp. as larval host plants (LHP), are usually classified as *M. arion arion*. In Italy, a more complex pattern is observed with three recognised subspecies. The first subspecies is *M. arion obscura* (Christoph, 1878), which is characterised by its small size and dark colours, is observed at high altitudes in the Alps and exploits *Thymus* spp. as LHP. The second, *M. arion ligurica* (Wagner, 1904), is bright blue in colour, inhabits low altitude grasslands, and exploits *Origanum vulgare* as its LHP. Finally, *M. a. arion* encompasses all other populations and *Thymus* spp. dependences. High variation in host ant use is observed in both countries, as immature butterflies were found with a total number of eight *Myrmica* species [14,21,24,25], Patricelli et al. unpublished observations.

Considering the presence of putative subspecies and the fact that Italian populations were potentially able to survive glaciations whereas Poland is a postglacial re-colonisation area, one should expect differences in the populations’ genetic structures. In this regard, we tested two competing hypotheses. The first hypothesis stated that “rear edge” populations (Italy) have retained higher genetic variability and are more differentiated from each other than Polish populations occurring in a recently colonised, more continuous, range; the second, the centre-periphery hypothesis [26,27], states that marginal populations (the Italian populations, in our case) should be genetically less diverse than those from the centre of the species’ distribution (Poland). Other additional goals of our studies were to examine possible genetic differentiation at a subspecies level and to try to identify potential evolutionarily significant units (ESUs) within this species. This may be interesting as Ugelvig [28] identified three haplotype groups in *Maculinea arion* and suggested that the lineages originated from different southern refugia during last glaciation. We sequenced the mitochondrial gene cytochrome oxidase subunit I (COI), known as the ‘barcoding gene’ [29], and the nuclear gene elongation factor 1α (EF-1α), which is a gene that is commonly used in phylogenetically oriented butterfly surveys to complement mtDNA markers e.g., [30,32]. Additionally, we tested our samples for the presence of *Wolbachia,* whose occurrence has been reported in several lycaenid butterflies including another representative of the genus *Maculinea* (i.e., *Maculinea alcon*) [33]. This endoparasitic bacterium is transmitted with the egg’s cytoplasm and may increase mtDNA differentiation among populations, due to heterogeneity of infections, or otherwise homogenise the host genetic structure, if all host populations are infected by the same strain [34]. The heterogeneity of infection may result in deep mitochondrial splits that are concordant with *Wolbachia* infection (Ritter et al. unpublished observations). Therefore, inspection of *M. arion* samples for *Wolbachia* is important for the interpretation of observed patterns because the presence of this endosymbiont can often confound the inference of the evolutionary history based on mtDNA data.

## Methods

### Sampling in Italy

**Figure 1:**
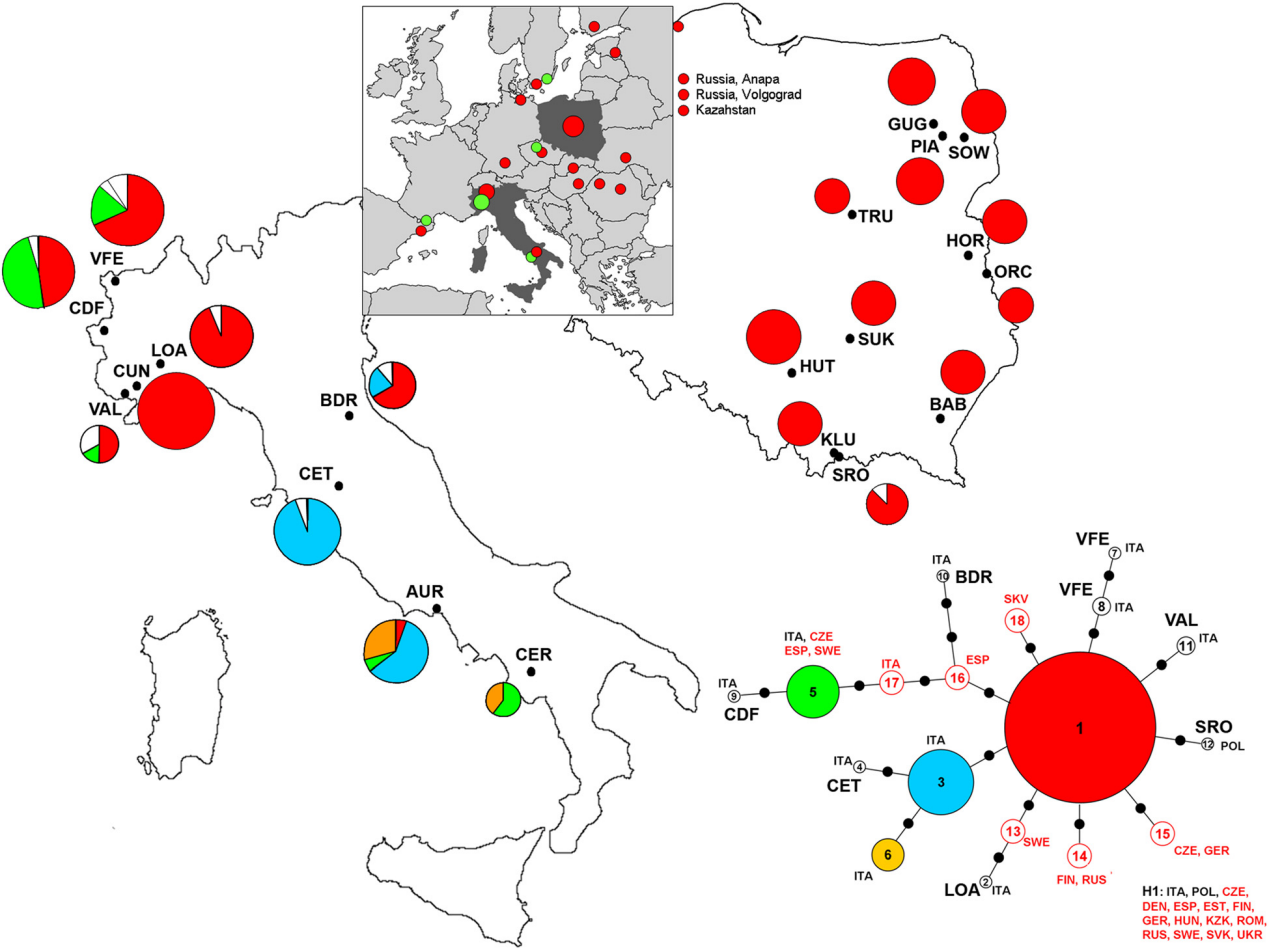
**Distribution of COI haplotypes among populations of *Maculinea arion *sampled in Italy and Poland.** The data are supplemented by haplotypes detected by Ugelvig [28] and Als et al. [58] and the maximum parsimony networks for all haplotypes. Unique COI haplotypes (present in individual populations only) are left uncoloured. The sizes of the circles are directly proportional to the number of individuals analysed. For full site names and other details see Table 1.

We sampled nine populations that were separated by distances between a minimum of 42 km between VAL and CUN and a maximum of 920 km between VFE and CER (Table 1, Figure 1). In the 2009 and 2010 seasons, a total of 153 of *M. arion* adults were captured, and a middle left leg was removed from each individual. After this procedure, the butterflies were immediately released, and the collected legs were stored in 99.9% ethanol. For every population, 5 to 25 samples were obtained.

**Table 1:** **Information on sampling locations of *Maculinea arion *studied in Italy and Poland**

**Locality**		**Region**	**Coordinates**	**Elevation (a.s.l.)**	**LHP**
**Code**	**Name**				
**Italy**					
VFE	Val Ferret	Aosta Valley	45 50’N/6 59’E	1636 m	*T. pulegioides*
CUN	Cuneo	Piedmont	44 25’N/7 35’E	443 m	*O. vulgare*
LOA	Loazzolo	Piedmont	44 39’N/8 14’E	356 m	*O. vulgare*
CDF	Colle Delle Finestre	Piedmont	45 04’N/7 03’E	2185 m	*Thymus* sp.
VAL	Valdieri	Piedmont	44 16’N/7 23’E	1400 m	*Thymus* sp.
BDR	Bagno di Romagna	Emilia Romagna	43 50’N/11 53E’	600 m	*O. vulgare*
CET	Mt Cetona	Tuscany	42 56’N/11 52’E	1006 m	*Thymus* sp.
AUR	Mt Aurunci	Lazio	41 18’N/13 38’E	1218 m	*Thymus* sp.
CER	Mt Cervati	Campania	40 19’N/15 25’E	1000 m	*Thymus* sp.
**Poland**					
GUG	Gugny	Biebrza Basin	53 19’N/22 35’E	100 m	*T. serpyllum*
PIA	Piaski	Narew Valley	53 13’N/22 45’E	105 m	*T. serpyllum*
SOW	Sowlany	Podlaise	53 09’N/23 15’E	160 m	*T. serpyllum*
TRU	Truskaw	Mazovia	52 19’N/20 46’E	80 m	*T. serpyllum*
HOR	Horodyszcze	Polesie	51 46’N/23 12’E	150 m	*T. serpyllum*
ORC	Włodawa	Polesie	51 31’N/23 35’E	150 m	*T. serpyllum*
HUT	Hutki-Kanki	Kraków-Częstochowa Upland	50 24’N/19 30’E	360 m	*T. serpyllum*
SUK	Suk	Kielce Upland	50 47’N/20 42’E	250 m	*T. serpyllum*
KLU	Kluszkowce	Gorce Mts.	49 27’N/20 19’E	730 m	*T. pulegioides*
SRO	Sromowce	Pieniny Mts.	49 24’N/20 24’E	530 m	*T. pulegioides*
BAB	Babice	Dyn Foothills	49 49’N/22 30’E	250 m	*T. pulegioides*

a.s.l. – above sea level. LHP – larval host plant.

Sampling sites were divided into three categories: woodland clearings on upland localities (350 - 600 m above see level) where *M. a. ligurica* exploits *Origanum vulgare* as LHP (CUN, LOA, BDR); mountain pastures in the Alps (1400 - 2200 m a. s. l.) where *M. a. obscura* is dependent on *Thymus* spp. (VAL, VFE, CDF); and clearings or pastures in the Apennines (1000 - 1200 m a. s. l.) where *M. a. arion* uses *Thymus* spp. (AUR, CER, CET) (Table 1, Figure 1).

### Sampling in Poland

We used DNA isolated from the legs or thoraxes of specimens previously collected for microsatellite analyses [35,36]. A total of 110 samples were analysed; all were from the main areas of *M. arion*’s distribution in the country and the sampling included eleven Polish populations. The distances between the Polish populations ranged from 8 km at a minimum (KLU and SRO) to a maximum of 460 km (GUG and SRO). The localities can be divided into two types: dry grasslands (often clearings in pine forests) on sandy flat areas where *Thymus serpyllum* was used as LHP and xerothermic grasslands on southerly exposed slopes with *T. pulegioides* (Table 1, Figure 1).

### DNA extraction and sequencing

Genomic DNA was extracted using a Genomic Mini Kit (A&A Biotechnology, Gdańsk, Poland). The Ron and Hobbes primers were used for the COI mtDNA fragment amplification, and ef51.9 and efrcM4 primers were used for the EF-1α gene [37]. PCR was performed on a GeneAmp PCR System 9700 (Applied Biosystems, Foster City, CA, USA) using a 5 μL reaction volume containing multiplex PCR Master Mix (1x) (Qiagen, Hilden, Germany), 0.2 μm of each primer, approximately 10-20 ng of genomic DNA and RNase-free water. Each PCR consisted of an initial activation step at 95°C for 15 min; 40 - 45 cycles of denaturation at 94°C for 30 s, annealing at 57°C for 90 s and extension at 72°C for 60 s; and a final extension at 60°C for 30 min. Direct sequencing was performed using the BigDye™ Terminator Cycle Sequencing Ready Reaction Kit 3.1 (Applied Biosystems). Both primers were used for sequencing both strands. The sequencing products were run on an ABI PRISM 3130 capillary automated sequencer (Applied Biosystems).

In addition, PCR to amplify the 16S rDNA to test for the presence of *Wolbachia* in *M. arion* was performed with the same amplification protocols using the W-Specf and W-Specr primers [38]. We screened all sampled populations, choosing 2 to 3 randomly selected individuals from each.

### Statistical analyses

DNA sequences were aligned in BioEdit v 7.0.4 [39] and revised manually. Haplotype reconstruction of the nuclear EF-1α gene from genotype data was conducted using the algorithms provided in PHASE as implemented in DnaSP 5.0 [40]. The number of haplotypes (*N*_*h*_), haplotype diversity (*h*), nucleotide diversity (*π*), and the number of segregating sites were calculated using ARLEQUIN 3.11 [41]. Relationships among the COI and the EF-1α haplotypes were represented as a haplotype network obtained by the statistical parsimony method using the TCS software [42]. Population divergence estimates were obtained as *F*_*ST*_ values in ARLEQUIN 3.11 [41]. The significance of pairwise *F*_*ST*_ values for the COI and the EF-1α genes was ascertained by 1000 permutations. The isolation by distance (IBD) pattern was tested by comparing the genetic differentiation between populations, as measured by pairwise *F*_*ST*_/(1- *F*_*ST*_), to the logarithm of geographical distance using IBD software [43]. Analysis of molecular variance (AMOVA) [44] was performed using ARLEQUIN 3.11 [41] (with 10000 permutations) to assess structuring within the data, and sampling sites were grouped as a single population (using *Φ*_*ST*_). To explore patterns of genetic divergence in more detail, we applied the spatial AMOVA procedure using SAMOVA ver. 1.0 [45]. This allowed us to identify the groupings of sampling sites that maximised the *F*_*CT*_ value based upon 10000 simulated annealing steps.

To test the phylogeographic structuring of haplotype distributions, we compared the average *G*_*ST*_ values (based on haplotype frequencies) to *N*_*ST*_ (based on haplotype frequencies and distance between haplotypes), as described by Pons and Petit [46], using DnaSP 5.10.01 [39].

## Results

### Sequence polymorphism at the COI mitochondrial gene

Mitochondrial DNA sequences (COI) were obtained for the 804-bp fragment in 225 individuals. We found twelve (eight singletons) haplotypes (GenBank accession nos. KC316050 - KC316061), which were defined by thirteen variable sites (eleven transitions and two transversions). The haplotypes differed by one to five substitutions. Possible relationships between the COI mtDNA haplotypes were estimated as a network, as shown in Figure 1. The network exhibited a star-shaped topology with H1 being the ancestral haplotype. This haplotype was the most common and most widespread, found in 18 out of 20 populations studied and only absent in two Italian sites (CER and CET). Generally, eleven COI haplotypes were present in Italy, ranging from one (CUN) to four (AUR and VFE) in any given population. However, all but one Polish population of *M. arion* were fixed for the same haplotype (H1). Moreover, H1 was the only haplotype shared between the two countries (Table 2 and Additional file 1: Table S1).

**Table 2:** **Genetic variation in *Maculinea arion *populations from Italy and Poland**

**Population**	**COI**			**EF-1α**		
	***N***	***h *(*N***_***h***_**)**	***π *(%)**	***N***	**(*N***_***h***_**)**	***π *(%)**
CER	5	0.600 (2)	0.38	5	0.467 (2)	0.10
AUR	17	0.596 (4)	0.13	20	0.396 (6)	0.10
CET	18	0.111 (2)	0.02	21	0.182 (4)	0.04
BDR	9	0.556 (3)	0.13	14	0.434 (5)	0.10
LOA	16	0.125 (2)	0.03	17	0.724 (6)	0.40
CUN	24	0.000 (1)	0.00	25	0.610 (3)	0.32
VAL	6	0.733 (3)	0.19	6	0.313 (4)	0.47
CDF	21	0.571 (3)	0.20	21	0.571 (7)	0.23
VFE	22	0.515 (4)	0.16	24	0.448 (4)	0.14
GUG	9	0.000 (1)	0.00	14	0.490 (3)	0.11
SOW	8	0.000 (1)	0.00	10	0.337 (2)	0.07
PIA	9	0.000 (1)	0.00	10	0.521 (2)	0.11
TRU	5	0.000 (1)	0.00	7	0.604 (3)	0.14
HOR	8	0.000 (1)	0.00	8	0.608 (3)	0.16
ORC	5	0.000 (1)	0.00	10	0.631 (6)	0.21
SUK	8	0.000 (1)	0.00	11	0.667 (5)	0.21
HUT	12	0.000 (1)	0.00	12	0.743 (8)	0.30
BAB	8	0.000 (1)	0.00	10	0.568 (3)	0.21
KLU	7	0.000 (1)	0.00	10	0.563(3)	0.13
SRO	8	0.250 (2)	0.06	8	0.675 (3)	0.31
**Total**	**225**	**0.480 (12)**	**0.12**	**263**	**0.635 (30)**	**0.23**

*N* – sample size, *h* – haplotype and gene diversity, number of haplotypes (*N*_*h*_) is given in parentheses, *π* – nucleotide diversity. COI – *cytochrome oxidase* subunit I (mtDNA), EF-1α – *elongation factor* (nuclear gene). For full site names and other details see Table 1.

For the pooled samples, the average COI mtDNA nucleotide (*π*) and haplotype (*h*) diversity values were estimated at 0.12% and 0.480, respectively. In the Italian populations, the average *π* and *h* were 0.17% and 0.651, respectively. For *M. arion* in Poland, the corresponding values were much smaller (*π* = 0.003%, *h* = 0.024) because the polymorphism was found only in the SRO population that had only two haplotypes (one being a singleton) (Table 2 and Additional file 1: Table S1). For the populations studied, *π* ranged from 0.00% to 0.38 and *h* ranged from 0.000 to 0.733 (Table 2).

All samples, regardless of the population origin, were positive for *Wolbachia* by PCR analysis. Our 16S rDNA sequences (334-bp, GenBank accession no KC337261 - KC337262) differed by a single substitution from the *Wolbachia* sequence found in the chrysomelid beetle *Diabrotica virgifera*[47] and therefore can be assigned to supergroup A with the highest probability.

### Sequence polymorphism at the EF-1α nuclear gene

**Figure 2:**
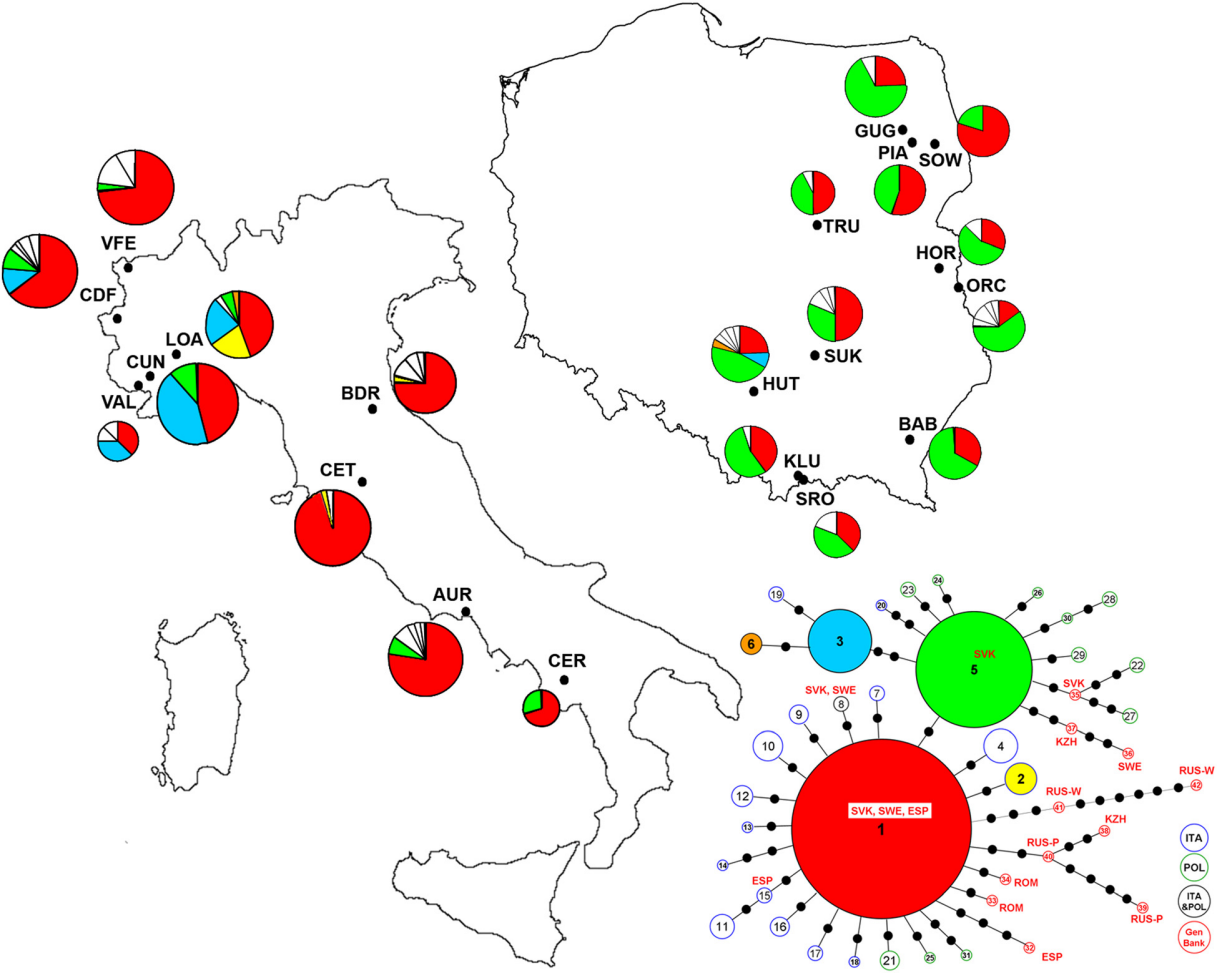
**Distribution of EF-1α haplotypes among populations of *Maculinea arion *sampled in Italy and Poland.** The data are supplemented by haplotypes detected by Als et al. [58] and the maximum parsimony networks for all haplotypes. Rare EF-1α haplotypes (present only in one or two populations) are left uncoloured. The sizes of the circles are directly proportional to the number of individuals analysed. For full site names and other details see Table 1.

Nuclear DNA sequences (EF-1α) were obtained by comparing a 460-bp fragment from 263 individuals. Altogether, 26 segregating sites (consisting of 29 substitutions, 20 transitions and 9 transversions) comprised the 30 haplotypes (17 singletons) (GenBank accession nos. KC316062 - KC316091) in the combined samples. Of these 30 haplotypes, the Italian populations contained 18 (4.56 haplotypes per population on average) and the Polish contained 17 (average per population 3.73) Table 2 and Additional file 1: Table S2). The possible relationships between the EF-1α haplotypes were estimated using a network, shown in Figure 2. The network exhibited a star-shaped topology, with H1 and H5 being the two ancestral haplotypes. The EF-1α haplotypes differed from one to six substitutions.

Only five out of the 30 EF-1α haplotypes (17%) were shared between the two countries. For all populations studied, the haplotype numbers ranged from two in the Mt. Cervati (CER), Sowlany (SOW) and Piaski (PIA) populations, up to seven in the Colle delle Finestre (CDF) populations and eight in the Hutki Kanki (HUT) population (Table 2 and Additional file 1: Table S2). The average *π* for the pooled sample was 0.21% (range: 0.02% - 0.47) and haplotype diversity was 0.632 (range: 0.094 – 0.743, Table 2). For Italy, the average *π* and *h* were 0.22% and 0.542, respectively. For *M. arion* in Poland, the corresponding values were similar (0.19% and 0.609, respectively).

### Genetic differentiation among the M. arion populations in Italy and Poland

**Figure 3:**
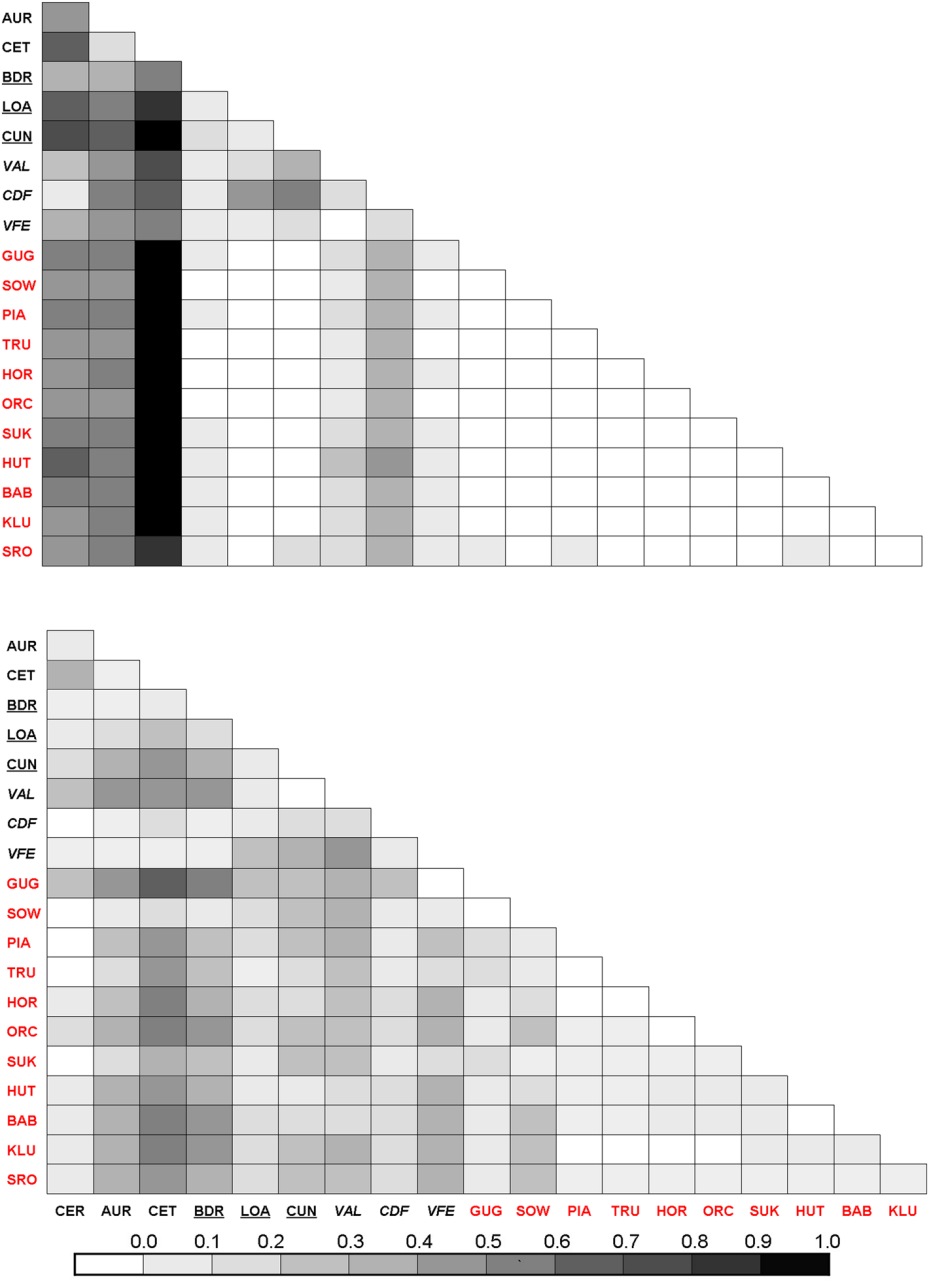
**Matrix of pairwise *F***_***ST.***_ Upper and lower matrices show the *F*_*ST*_ values for COI gene and for EF-1α, respectively, between each population; localities are provided in Table 1. Abbreviations of populations from Italy and Poland are given in black and red, respectively and additionally localities of *M. arion ligurica* and *M. obscura* are underlined and italised, respectively.

Based upon the COI genetic data, there was a statistically significant and high degree of genetic differentiation among the 20 *M. arion* populations studied (*F*_*ST*_ = 0.436, *P* < 0.001). The pairwise *F*_*ST*_ values between the populations were highly variable and ranged from 0.000 to 0.955 (Figure 3, Additional file 1: Table S3). In Italy, we observed very high and statistically significant population differentiation (average *F*_*ST*_ = 0.432, *P* < 0.001; range: 0.000 – 0.955). However, in Poland, the average genetic differentiation at the COI gene among all the populations was very low and not statistically significant (*F*_*ST*_ = 0.005, *P* > 0.05; range: 0.000 - 0.054).

The average genetic differentiation among all 20 populations of *M. arion* for the EF-1α gene was also high (*F*_*ST*_ = 0.179) and statistically significant (*P* < 0.001). The pairwise *F*_*ST*_ values ranged from 0.000 to 0.678. The average pairwise *F*_*ST*_ value between Italian populations was high and statistically significant (*F*_*ST*_ = 0.176, *P* < 0.001; range: 0.000 – 0.499). Moderate and significant, genetic differentiation was found among the Polish populations (average *F*_*ST*_ = 0.059; *P* < 0.001; range: 0.000 – 0.392) (Figure 3, Additional file 1: Table S3). No pattern of isolation by distance (IBD) was detected among Italian populations of *M. arion*, with respect to either the COI gene (*r*^2^ = 0.02, *P* > 0.05) or for the EF-1α gene (*r*^2^ = 0.03, *P* > 0.05) nor among Polish populations with respect to the EF-1α gene (*r*^2^ = 0.01, *P* > 0.05). The IBD analysis was not performed on the Polish samples for the COI gene because the majority of pairwise *F*_*ST*_ values were zero.

The geographical structuring among the *M. arion* haplotypes was highly supported by AMOVA results, for which all sampling sites were treated as a single group (*Φ*_*ST*_ = 0.438, *P* < 0.001 for the mtDNA COI gene; *Φ*_*ST*_ = 0.195, *P* < 0.001 for the EF-1α gene). When the Italian and Polish samples were treated as two separate groups, the AMOVA was not significant for the COI gene (*Φ*_*CT*_ = 0.073, ns). However, significant structuring between Italy and Poland was found for the EF-1α gene (*Φ*_*CT*_ = 0.122, *P* < 0.01). In Italy, no significant structuring was observed with respect to the LHP for either marker (mtDNA: *Φ*_*CT*_ = 0.089, ns; EF-1α: *Φ*_*CT*_ = 0.054, ns). The same result was obtained when the Italian populations were regrouped as lowland (up to 600 m) and highland (over 600 m).

SAMOVA was used separately on the Italian and Polish populations to identify which subdivision was most likely to explain the genetic structure observed in *M. arion* for the COI and EF-1α genes. For the COI gene in Italy, the data were best explained by assuming four groups of populations. The first group consisted of the AUR and CET samples; group two included populations from the BDR, LOA, CUN, VAL and VFE; and groups three and four were represented by the CER and the CDF populations, respectively. The percentage of variation among these four groups was high at 48.43% (*P* < 0.001); among the populations within the groups, the variation was 3.26% (*P* < 0.001), and within the populations, the variation was 48.31% (*P* < 0.001). The maximum percentage of variation for the EF-1α gene in Italy (28.34%, *P* < 0.05) occurred under the assumption of two population groups (CUN and VAL compared to all other Italian samples), while the variation among populations within groups was 5.37% (*P* < 0.001) and within populations was 66.29% (*P* < 0.001). The analysis of the Polish samples revealed no grouping of populations, and the EF-1α gene data were best explained by assuming a single population group (*Φ*_*ST*_ = 0.059, *P* < 0.001). No such analysis was performed for the mtDNA on Polish samples because of the almost complete lack of polymorphism.

For the EF-1α gene, the average *N*_*ST*_ value (0.156) was significantly (*P* < 0.001) higher than the average *G*_*ST*_ value (0.076) among 20 populations studied, indicating a phylogeographic structuring of *M. arion* haplotype distributions. The same pattern was also found when the Italian samples (*N*_*ST*_ = 0.143, *G*_*ST*_ = 0.050; *P* < 0.001) and the Polish samples (*N*_*ST*_ = 0.056, *G*_*ST*_ = 0.026; *P* < 0.001) were analysed separately. Interestingly, both the *N*_*ST*_ and *G*_*ST*_ values for the EF-1α gene were significantly higher among Italian samples than among Polish ones (*P* < 0.001). No phylogeographically significant structuring was found for the mtDNA in Italy (*N*_*ST*_ = 0.319, *G*_*ST*_ = 0.262; ns).

## Discussion

### Rear edges versus continuous areas of distribution in M. arion

Perfectly matching rear edge theory [7], this study revealed great genetic differentiation among *M. arion* Italian populations, both at the COI mtDNA and the nuclear EF-1α genes. This is in contrast to the almost total absence of mtDNA polymorphism and the low to moderate genetic differentiation at the nuclear gene found for *M. arion* populations inhabiting Poland, a recently deglaciated area. Moreover, the rear edge populations (Italy) possessed as many as 11 out of the 12 mtDNA haplotypes recorded in this study; thereby, emphasising their relevance in the context of biodiversity conservation and, most importantly, from the evolutionary perspective.

The mtDNA diversity in Italy may be thus an example of 'southern richness’ as opposed to ‘northern purity' as defined by Hewitt [1,3,48], e.g. high polymorphism and divergence in the south and low variation and lack of divergence in the north. “Southern richness” was also visible for the nuclear gene EF-1α as the average number of alleles per population and the genetic differentiation among them was significantly larger than among Polish populations.

Thus, our data do not support the alternative scenario that suggests lower genetic diversity in populations at the edge of a species’ distribution compared to that in the centre [26,27]. The high genetic diversity found in Southern Europe can be attributed to prolonged demographic stability in the populations occurring in these areas [1] and by the high geographic structuring of populations evolving in allopatric conditions [49,50]. In Italy, prolonged demographic stability was assumed to create a common history for many taxa and, recently, allopatric differentiation (refugia-within-refugia) was described in *Bombina pachypus*[51]. The identification of four distinct genetic groups for mtDNA as well as similar *N*_*ST*_ and *G*_*ST*_ values emphasize the role of the genetic drift as the main force responsible for the differentiation among Italian populations. In Italy, we found more structuring and greater genetic differentiation for the COI gene than for the EF-1α gene. Mitochondrial markers are characterised by much faster lineage sorting rates and more frequent haplotype extinctions than the bi-parental nuclear markers. Consequently, as is widely recognised for many Lepidoptera e.g., [52,53], differentiation in mtDNA among populations is usually much stronger than in nuclear DNA (e.g., EF-1α).

### Population structure and colonisation patterns

Although our data on mitochondrial genetic material suggested a significant population genetic structure, they did not support the subdivision of the populations into macrogeographic groups (Poland versus Italy). The most common mtDNA haplotype in our samples (H1) is indeed the most widespread throughout the species’ range because it was recorded also from Catalonia, Germany, Denmark, Sweden, Estonia, Finland, the Czech Republic, Slovakia, Romania, Hungary, Ukraine, Russia and Kazakhstan. Moreover, the second most common haplotype (H5), which we found in five Italian sites (CER, AUR, VAL, CDF, VFE), is known to occur in Catalonia, the Czech Republic and Sweden [28]. As a result, our mtDNA data do not fully support the colonisation pattern hypothesized by Ugelvig [28]. The author suggests the existence of three different genetic groups in the Western Palaearctic originating from different southern refugia in the Iberian Peninsula, the Balkans and Asia and thus three postglacial colonisation routes. The Alps are known to have acted as an initial barrier to the possible expansion of Italian genetic pools towards Northern Europe for many species [3,9], but our data do not allow us to exclude Italy as a possible source area for Central and Northern European *M. arion* populations. In Poland, we almost exclusively found a single haplotype, which was also the most frequent in northern Italy (H1). Of course, a “European” origin of north Italian populations cannot be ruled out either. This would mean that the dispersion from the East has reached northern Italy. Our results have indeed shown that the ancestral haplotype (H1) is dominant in northern Italy, but is rare (AUR) or absent (CET, CER) in central and southern Italy. Ugelvig [28] suggests that, in the case of *M. arion*, Italy was colonised from Iberia. It should be noted, however, that only two samples from Italy were analysed, while our extensive study showed that Italy was rather colonised from two different routes.

We detected three unique and closely related mtDNA haplotypes (H3, H4, H6) in southern Italy. A similar pattern was found in Italy for the *Euscorpio carpathicus* complex, where Salomone et al. [54] concluded that the presence of ancestral haplotypes in the northern part of the country meant that other refugia, outside of the Apennine, may have existed for these taxa. Possible sources are the Balkans, which northern Italy was connected to by the Adriatic bridge during the quaternary cold periods [2], and the Iberian Peninsula perhaps via Col di Tenda, as was the case for the western lineage of *Melanargia galathea*[55]. However, both directions (i.e., also from Italy towards Iberia) are possible, as it was suggested for *Polyommatus coridon*[5]. This conclusion is supported by our data showing the presence of one haplotype (H5) in Catalonia, in the Alpes Maritimes (VAL is 20 km away from Col di Tenda) and all along the Italian latitudinal gradient.

On the other hand, the presence of unique haplotypes in southern Italy concurs with the hypothesis that, contrary to common belief, most relic rear edge populations may not have been the source of major postglacial re-colonisations, thereby preserving their high genetic distinctiveness [7]. Rear edges, as ‘relic hotspots’, may be considered as regions of special conservation concern, as they often coincide with centres of high biodiversity and endemism. To fully resolve *M. arion* phylogeographical pattern further studies should be based on a much higher number of specimens, especially coming from other South European areas as well as central populations.

*M. arion* showed moderate to high levels of diversity in the nuclear gene EF-1α. Altogether, 30 haplotypes were found, 18 occurring in Italy and 17 in Poland. Interestingly, a study carried out on *M. alcon* in a closely overlapping area found only six haplotypes among the 179 individuals sampled [32]. Our results from the EF-1α gene support the allozyme and microsatellite data [56,57] by showing that *M. arion* exhibits a rather high nuclear genetic variability when compared to other members of the genus *Maculinea*[33,35,58]. It has been suggested that the specialised parasitic life style of *Maculinea* butterflies makes them prone to genetic drift and therefore to a loss in genetic variation [58]. However, *M. arion* is considered a less advanced social parasite because it preys on ant larvae and shows a lower level of host ant specificity. In this case, it is possible that the effective population size of *M. arion* is higher than in other members of the genus, especially *M. alcon* and *M. ‘rebeli’*. In this regard, it is interesting to observe that recent genetic data obtained on Danish and Swedish populations suggest that *M. arion* is more dispersive and less sensitive to genetic erosion than was previously assumed [59,60].

The AMOVA analysis of nuclear genes, through EF-1α, showed significant genetic differentiation between the Italian and Polish populations. This may indicate a different population origin occurred in both countries and some indications of this can be found among 24 sequences available in Genbank. A total of twelve EF-1α haplotypes in addition to our samples (30 haplotypes) were deposited by Als et al. [58]. In the pooled sample, the most common haplotype (H1) was shared by all populations in Poland and Italy and has been found in Slovakia, Sweden and Spain. The H5 haplotype was the most common haplotype throughout Poland and network suggests its expansion here. It was also present in six out of nine Italian populations, although never as a dominant haplotype, and has been identified in Slovakia and Sweden as well. This suggests that Poland was not colonised exclusively from northern Italy. Poland and Italy shared five out of the 30 EF-1α haplotypes and one of them (H8) was also found in Slovakia [58]. The presence of two common haplotypes in Poland may indicate that this part of Europe was colonised from two different routes (from the east and west). Finally, the H15 haplotype, which we identified only in northern Italy (CDF), was also found in Spain. The latter finding (and the presence of H1 in both countries that seems to be ancestral for the majority of Italian haplotypes; see network), may corroborate the existence of relationships between the Iberian and the Italian peninsula, as indicated by mtDNA and postulated by Ugelvig [28].

The low to moderate population differentiation observed in Poland for the EF-1α gene concurs with results from a previous study involving microsatellite markers that was conducted on the same DNA material; in which, rather low levels of differentiation for the species were determined, although some populations were highly isolated and the genetic differentiation was indicated to be mainly shaped by habitat fragmentation [36]. Interestingly one population (HUT), where suitable biotope for *M. arion* was extensive, yielded the highest level of polymorphism in both EF-1α and microsatellite markers [36].

We did not find any evidence for IBD for any population grouping or for molecular marker. The lack of any correlation between geographic and genetic distances can be explained by historical demographic processes or cycles of colonisation and extinction. A similar pattern was identified for some Apennine populations of *Bombina pachypus* by Canestrelli et al. [51].

### Putative subspecies divisions

Our genetic data do not support any subspecies divisions in *M. arion*. Among the Italian samples there were representatives of both *M. a. ligurica* (LOA, CUN, BDR) and *M. a. obscura* (VFE, CDF, VAL), while the remaining Italian populations (CET, AUR, CER), as well as all Polish populations belonged to *M. a. arion.* The analysis of the bar code sequence showed that two of the three populations belonging to *M. arion ligurica* were characterised by fixed or almost fixed haplotype H1, which was also fixed in 10 of 11 Polish populations and almost fixed in the remaining population. No species or subspecies-level differentiation was detected also as far as EF-1α gene is concerned. Hence, the results of our study are in agreement with the allozyme analysis of Hungarian populations indicating no differences between *Origanum* and *Thymus* dependent populations [57]. This suggests that, despite the high ecological and morphological variability which led to the description of several subspecies, *M. arion* is a species with an absence of deep historic population isolation. Sielezniew & Dziekańska [61] hypothetise that clinal adaptation is a more likely explanation for the observed wing pattern variation in this butterfly than incipient speciation.

It is worth noting that in the case of two other *Maculinea* taxa (*M. alcon* and *M. ‘rebeli’*), molecular analysis revealed no differences supporting division into separate species [33,62] and possible selective sweeps cannot be ruled out as well [33]. A lack of host-associated genetic differentiation in the barcoding gene COI is reported by Craft et al. [63] from New Guinea, where 7 out of 28 analysed Lepidoptera species exhibited the same haplotypes despite the use of multiple LHPs. A similar pattern was observed by Hulcr et al. [64] in the moth *Homona mermerodes.*

We can speculate that use of different LHPs by a locally monophagous butterfly species, such as *M. arion*, has not been a mean to promote speciation, but can be ascribed to local adaptations in the species’ phenology that have evolved recently. For instance, when one of the glacial refugia was localised at the southern base of the Alps, after deglaciation, some populations could expand their range to higher altitudes tracking hosts re-colonisation, while others were able to survive in the lowlands by shifting to a novel host plant [11]. If this scenario is true, the *M. a. ligurica* populations may represent a relic of *M. arion* populations that survived at the base of the Alps and were a source for the re-colonisation of the Alps.

### No effects of Wolbachia infection on population differentiation in M. arion

All the populations in this study were infected by *Wolbachia*, and screening of all our samples suggested 100% prevalence of a single strain of the bacterium (supergroup A). The most common effect of a *Wolbachia* infection is cytoplasmic incompatibility (CI), which results in infected males that are unable to successfully reproduce with either uninfected females or females infected by a different strain. As a consequence, the distribution of mtDNA variation in *Wolbachia* infected populations may not conform to the expectations of the neutral theory [34,65], even though endosymbiotic infections may not explain the divergent haplotype groups within some butterfly populations [66]. The star-like network of haplotypes found in our study is typical for a rapid expansion model [67], but could also be shaped by selective sweeps of *Wolbachia*, as hypothesised for *Polygonia c-album*[68].

The congeneric *Maculinea alcon* (including the xerothermophilous ecotype ‘*M. rebeli’*) is also reported to have *Wolbachia* infection [33]*.* However, a different strain (supergroup B) was detected in samples of this species collected across Poland and Lithuania. Sielezniew et al. [33] hypothesise that a selective sweep could have reduced mtDNA diversity in the past, as only a single COI mtDNA haplotype was found in that area. Interestingly, our study indicates great genetic differentiation for mtDNA among studied populations in Italy, all infected by the same strain of the bacterium. This could mean that *Wolbachia* is not responsible for the observed pattern of genetic differentiation among the studied *M. arion* populations. It is probable that the *Wolbachia* infection in *M. arion* populations preceded both the expansion from glacial refugia and ecological specialisation.

## Conclusions

Our study shows that the rear edge theory may explain the observed differences in the genetic differentiation pattern in *M. arion* between the two distant parts of the European species range. Although we were unable to demonstrate any evolutionarily significant units, we suggest that Italy is an example of a rear edge area and should be considered as a region of special conservation concern, and therefore important for retaining genetic diversity in *M. arion*. However, the lack of correlation between the genetic differentiation and any subspecies divisions or ecological variation indicate that the observed specialisations are relatively recent in origin. We also demonstrate that the patterns of mtDNA diversity found in Poland and Italy cannot be explained by endosymbiotic infection and results from the colonisation patterns as well as genetic drift.

## Competing interests

The authors declare that they have no competing interests.

## Authors' contributions

DP, MS, MR and EB designed the study. DP, MS, SB, FB and MW carried out field work. DPT, MR, DP, MB and RR performed laboratory work, genetic and statistical analyses. MS, MR, DP and RR drafted the manuscript. SB, FB, MW and EB critically reviewed the manuscripts. All authors read and approved the final manuscript.

## Supplementary Material

Additional file 1 **Table S1.** Haplotype frequencies of the COI mtDNA gene in nine populations of *M. arion* from Italy and eleven populations from Poland. **S2.** Haplotype frequencies of the EF-1α nuclear gene in nine populations of *M. arion* from Italy and eleven populations from Poland. **S3.** Genetic differentiation (pairwise *F*_*ST*_ main text and supplementary table s3) at the COI gene (listed above the diagonal) and for EF-1α (listed below the diagonal) among nine populations of *M. arion* in Italy and eleven populations of *M. arion* in Poland (for full names of the locations see Tab. 1)*.* Any significant *F*_*ST*_ main text and supplementary table s3 values are given in bold. Click here for file

## References

[B1] Hewitt GM Some genetic consequences of ice ages, and their role, in divergence and speciation Biol J Linn Soc 1996 58 247 276

[B2] Taberlet P Fumagalli L Wust-Saucy AG Cosson JF Comparative phylogeography and postglacial colonization routes in Europe Mol Ecol 1998 7 453 464 10.1046/j.1365-294x.1998.00289.x 9628000

[B3] Hewitt GM Post-glacial re-colonization of European biota Biol J Linn Soc 1999 68 87 112 10.1111/j.1095-8312.1999.tb01160.x

[B4] Hewitt GM Genetic consequences of climatic oscillations in the Quaternary Philos T R Soc B 2004 359 183 195 10.1098/rstb.2003.1388 PMC1693318 15101575

[B5] Schmitt T Molecular Biogeography of Europe: Pleistocene cycles and Postglacial trends Front Zool 2007 4 11 10.1186/1742-9994-4-11 17439649 PMC1868914

[B6] Besold J Schmitt T Tammaru T Cassel-Lundhagen A Strong genetic impoverishment from the centre of distribution in southern Europe to peripheral Baltic and isolated Scandinavian populations of the pearly heath butterfly J Biogeogr 2008 35 2090 2101 10.1111/j.1365-2699.2008.01939.x

[B7] Hampe A Petit RJ Conserving biodiversity under climate change: the rear edge matters Ecol Lett 2005 8 461 467 10.1111/j.1461-0248.2005.00739.x 21352449

[B8] Petit RJ Aguinagalde I de Beaulieu JL Bittkau C Brewer S Cheddadi R Ennos R Fineschi S Grivet D Lascoux M Mohanty A Muller-Starck G Demesure-Musch B Palme A Martın JP Rendell S Vendramin GG Glacial refugia: hotspots but not melting pots of genetic diversity Science 2003 300 1563 1565 10.1126/science.1083264 12791991

[B9] Bilton DT Mirol PM Mascheretti S Fredga K Zima J Searle JB Mediterranean Europe as an area of endemism for small mammals rather than a source for northwards postglacial colonization Proc R Soc Lond B 1998 265 1219 1226 10.1098/rspb.1998.0423 PMC1689182 9699314

[B10] Schmitt T Biogeographical and evolutionary importance of the European high mountain systems Front Zool 2009 6 9 10.1186/1742-9994-6-9 19480666 PMC2700098

[B11] Bonelli S Barbero F Casacci LP Cerrato C Patricelli D Sala M Vovlas A Witek M Balletto E Grillo O, Verona G Butterfly Diversity in a Changing Scenario Changing Diversity in Changing Environment 2011 Rijeka: InTech 99 132

[B12] Thomas JA Pullin AS 1995: The ecology and conservation of *Maculinea arion* and other European species of large blue butterfly Ecology and Conservation of Butterflies 1995 London: Chapman and Hall 180 197

[B13] Thomas JA Simcox DJ Clarke RT Successful Conservation of a Threatened *Maculinea Butterfly* Science 2009 325 80 83 10.1126/science.1175726 19541953

[B14] Sielezniew M Dziekańska I Stankiewicz-Fiedurek AM Multiple host-ant use by the predatory social parasite *Phengaris* (=*Maculinea*) *arion* (Lepidoptera, Lycaenidae) J Insect Conserv 2010 14 141 149 10.1007/s10841-009-9235-0

[B15] Elmes GW Akino T Thomas JA Clarke RT Knapp JJ Interspecific differences in cuticular hydrocarbon profiles of *Myrmica* ants are sufficiently consistent to explain host specificity by *Maculinea* (large blue) butterflies Oecologia 2002 130 525 535 10.1007/s00442-001-0857-5 28547253

[B16] Barbero F Bonelli S Thomas JA Baletto E Schönrogge K Acoustical mimicry in a predatory social parasite of ants J Exp Biol 2009 212 4084 4090 10.1242/jeb.032912 19946088

[B17] Van Swaay CAM Warren MS Red Data Book of European Butterflies (Rhopalocera). In Nature and Environment Series no. 99 1999 Strasbourg: Council of Europe

[B18] Van Swaay C Cuttelod A Collins S Maes D Lopez Munguira M Šašić M Settele J Verovnik R Verstrael T Warren M Wiemers M Wynhoff I European Red List of Butterfies 2010 Luxembourg: Publications Office of the European Union

[B19] Randle Z Simcox DJ Schönrogge K Wardlaw JC Thomas JA Settele J, Kühn E, Thomas JA *Myrmica* ants as keystone species and *Maculinea* arion as an indicator of rare niches in UK grasslands Studies on the Ecology and Conservation of Butterflies in Europe Vol. 2 Species Ecologyalong a European Gradient: Maculinea Butterflies as a Model 2005 Sofia-Moscow: J. Pensoft 26 28

[B20] Spitzer L Benes J Dandova J Jaskova V Konvicka M The Large Blue butterfly, *Phengaris* [*Maculinea*] *arion*, as a conservation umbrella on a landscape scale: The case of the Czech Carpathians Ecol Indic 2009 9 1056 1063 10.1016/j.ecolind.2008.12.006

[B21] Casacci LP Witek M Barbero F Patricelli D Solazzo G Baletto E Bonelli S Habitat preferences of *Maculinea arion* and its *Myrmica* host ants: implications for habitat management in Italian Alps J Insect Conserv 2011 15 103 110 10.1007/s10841-010-9327-x

[B22] Tolman T Lewington R Collins Butterfly Guide: The Most Complete Field Guide to the Butterflies of Britain and Europe 2009 London: Harper Collins

[B23] Thomas JA Helsdingen PJ, Willemse L, Speight MCD *Maculinea arion* (Linnaeus, 1758) Background information on invertebrates of the Habitats Directive and the Bern Convention. Part I - Crustacea, Coleoptera and Lepidoptera - Nature and Environment No 79 1996 Strasbourg: Council of Europe Publishing 157 163

[B24] Sielezniew M Stankiewicz AM *Myrmica sabuleti* (Hymenoptera: Formicidae) not necessary for the survival of the population of *Phengaris* (*Maculinea) arion* (Lepidoptera: Lycaenidae) in eastern Poland: lower host-ant specificity or evidence for geographical variation of an endangered social parasite? Eur J Entomol 2008 105 637 641

[B25] Sielezniew M Patricelli D Dziekańska I Barbero F Bonelli S Casacci LP Witek M Baletto E The first record of *Myrmica lonae* (Hymenoptera: Formicidae) as a host of socially parasitic Large Blue butterfly *Phengaris* (*Maculinea*) arion (Lepidoptera: Lycaenidae) Sociobiology 2010 56 465 475

[B26] Lawton JH Range, population abundance and conservation Trends Ecol Evol 1993 8 409 413 10.1016/0169-5347(93)90043-O 21236213

[B27] Vucetich JA Waite TA Spatial patterns of demography and genetic processes across the species range: null hypotheses for landscape conservation genetics Conserv Gen 2003 4 639 645 10.1023/A:1025671831349

[B28] Ugelvig LV Ecological genetics and evolution of the Large Blue butterfly – consequences of an extraordinary lifecycle.PhD thesis 2010 Københavns Universitet, Biologisk Institut

[B29] Hebert PDN Cywinska A Ball SL DeWaard JR Biological identifications through DNA barcodes Proc R Soc B 2003 270 313 322 10.1098/rspb.2002.2218 PMC1691236 12614582

[B30] Wahlberg N Weingartner E Nylin S Towards a better understanding of the higher systematics of Nymphalidae (Lepidoptera: Papilionoidea) Mol Phyl Evol 2003 28 473 484 10.1016/S1055-7903(03)00052-6 12927132

[B31] Wahlberg N Brower AVZ Nylin S Phylogenetic relationships and historical biogeography of tribes and genera in the subfamily Nymphalinae (Lepidoptera: Nymphalidae) Biol J Linn Soc 2005 86 227 251 10.1111/j.1095-8312.2005.00531.x

[B32] Kodandaramaiah U Wahlberg N Phylogeny and biogeography of *Coenonympha* butterflies (Nymphalidae: Satyrinae) - patterns of colonization in the Holarctic Syst Entomol 2009 34 315 323 10.1111/j.1365-3113.2008.00453.x

[B33] Sielezniew M Rutkowski R Ponikwicka D Ratkiewicz M Dziekańska I Švitra G Differences in genetic variability between two ecotypes of endangered myrmecophilous butterfly *Phengaris* (=*Maculinea) alcon* – the setting of conservation priorities Insect Conserv Diver 2012 5 223 236 10.1111/j.1752-4598.2011.00163.x

[B34] Hurst GDD Jiggins FM Problems with mitochondrial DNA as a marker in population, phylogeographic and phylogenetic studies: the effects of inherited symbionts Proc R Soc Lond B 2005 272 1525 1534 10.1098/rspb.2005.3056 PMC1559843 16048766

[B35] Rutkowski R Sielezniew M Szostak A Contrasting levels of polymorphism in cross-amplified microsatellites in two endangered xerothermophilous, obligatorily myrmecophilous, butterflies of the genus *Phengaris* (*Maculinea*) (Lepidoptera: Lycaenidae) Eur J Entomol 2009 106 457 469

[B36] Sielezniew M Rutkowski R Population isolation rather than ecological variation explains the geneticstructure of endangered myrmecophilous butterfly *Phengaris*(=*Maculinea) arion* J Insect Conserv 2012 16 39 50 10.1007/s10841-011-9392-9

[B37] Monteiro A Pierce NE Phylogeny of *Bicyclus* (Lepidoptera: Nymphalidae) inferred from *COI, COII* and *EF1*-α gene sequences Mol Phyl Evol 2001 18 264 281 10.1006/mpev.2000.0872 11161761

[B38] Werren JH Windsor DM *Wolbachia* infection frequency in insects: evidence of a global equilibrium? Proc R Soc B 2000 267 1277 1285 10.1098/rspb.2000.1139 PMC1690679 10972121

[B39] Hall TA BioEdit: A user-friendly biological sequence alignment editor and analysis program for Windows 95/98/NT Nucleic Acids Symp Ser 1999 41 95 98

[B40] Librado M Rozas J DnaSP v5: a software for comprehensive analysis of DNA polymorphism data Bioinformatics 2009 25 1451 1452 10.1093/bioinformatics/btp187 19346325

[B41] Excoffier L Laval G Schneider S Arlequin ver. 3.0: An integrated software package for population genetics data analysis Evol Bioinform Online 2005 1 47 50 19325852 PMC2658868

[B42] Clement M Posada D Crandall KA TCS: A computer program to estimate gene genealogies Mol Ecol 2000 9 1657 1659 10.1046/j.1365-294x.2000.01020.x 11050560

[B43] Bohonak AJ IBD (Isolation by Distance): A Program for Analyses of Isolation by Distance J Hered 2002 93 153 154 10.1093/jhered/93.2.153 12140277

[B44] Excoffier L Smouse PE Quattro JM Analysis of molecular variance inferred from metric distances among DNA haplotypes – application to human mitochondrial DNA restriction data Genetics 1992 131 479 491 1644282 10.1093/genetics/131.2.479 PMC1205020

[B45] Dupanloup I Schneider S Excoffier L A simulated annealing approach to define the genetic structure of populations Mol Ecol 2002 11 2571 2581 10.1046/j.1365-294X.2002.01650.x 12453240

[B46] Pons O Petit RJ Measuring and testing genetic differentiation with ordered and unordered alleles Genetics 1996 144 1237 1245 8913764 10.1093/genetics/144.3.1237 PMC1207615

[B47] Giordano R Jackson JJ Robertson HM The role of *Wolbachia* bacteria in reproductive incompatibilities and hybrid zones of *Diabrotica* beetles and Gryllus crickets Proc Natl Acad Sci USA 1997 94 11439 11444 10.1073/pnas.94.21.11439 9326628 PMC23493

[B48] Hewitt GM The genetic legacy of the Quaternary ice ages Nature 2000 405 907 913 10.1038/35016000 10879524

[B49] Guillaume CP Heulin B Arrayago MJ Bea A Braña F Refuge areas and suture zones in the Pyrenean and Cantabrian regions: geographic variation of the female MPI sex-linked allele among oviparous populations of the lizard *Lacerta* (*Zootoca*) *vivipara* Ecography 2000 23 3 10 10.1111/j.1600-0587.2000.tb00255.x

[B50] Sanz N García-Marín JL Pla C Divergence of brown trout (*Salmo trutta*) within glacial refugia Can J Fish Aquat Sci 2000 57 2201 2210 10.1139/f00-199

[B51] Canestrelli D Cimmaruta R Costantini V Nascetti G Genetic diversity and phylogeography of the Apennine yellow-bellied toad *Bombina pachypus*, with implications for conservation Mol Ecol 2006 15 3741 3754 10.1111/j.1365-294X.2006.03055.x 17032271

[B52] Sielezniew M Ponikwicka D Ratkiewicz M Rutkowski R Dziekańska I Kostro-Ambroziak A Diverging patterns of mitochondrial and nuclear diversity in the specialized butterfly *Plebejus argus* (Lepidoptera: Lycaenidae) Eur J Entomol 2011 108 537 545

[B53] Zakharov EV Hellmann JJ Genetic differentiation across a latitudinal gradient in two co-occurring butterfly species: revealing population differences in a context of climate change Mol Ecol 2007 17 189 208 17784923 10.1111/j.1365-294X.2007.03488.x

[B54] Salomone N Vignoli V Frati F Bernini F Species boundaries and phylogeography of the “*Euscorpius carpathicus* complex” (Scorpiones: Euscorpiidae) in Italy Mol Phylogenet Evol 2007 43 502 514 10.1016/j.ympev.2006.08.023 17150377

[B55] Habel JC Schmitt T Müller P The fourth paradigm pattern of postglacial range expansion of European terrestrial species: the phylogeography of the Marbled White butterfly (Satyrinae, Lepidoptera) J Biogeogr 2005 32 1489 1497 10.1111/j.1365-2699.2005.01273.x

[B56] Pecsenye K Bereczki J Tihanyi B Tóth A Peregovits L Varga Z Genetic differentiation among the *Maculinea* species (Lepidoptera: Lycaenidae) in Eastern Central Europe Biol J Linn Soc 2007 91 11 21 10.1111/j.1095-8312.2007.00781.x

[B57] Bereczki JJ Tóth P Tóth A Bátori E Pecsenye K Varga Z The genetic structure of phenologically differentiated Large Blue (*Maculinea arion*) populations (Lepidoptera: Lycaenidae) in the Carpathian Basin Eur J Entomol 2011 108 519 527

[B58] Als TD Vila R Kandul NP Nash DR Yen SH Hsu YF Andre A Mignault AA Boomsma JJ Pierce NE The evolution of alternative parasitic life histories in large blue butterflies Nature 2004 432 386 390 10.1038/nature03020 15549104

[B59] Ugelvig LV Nielsen PS Boomsma JJ Nash DR Reconstructing eight decades of genetic variation in an isolated Danish population of the large blue butterfly *Maculinea arion* BMC Evol Biol 2011 11 201 10.1186/1471-2148-11-201 21745368 PMC3146443

[B60] Ugelvig LV Andersen A Boomsma JJ Nash DR Dispersal and gene flow in the rare, parasitic Large Blue butterfly *Maculinea arion* Mol Ecol 2012 21 3224 3236 10.1111/j.1365-294X.2012.05592.x 22548466

[B61] Sielezniew M Dziekańska I Geographical variation in wing pattern in *Phengaris* (=*Maculinea*) *arion* (L.) (Lepidoptera: Lycaenidae): subspecific differentiation or clinal adaptation? Ann Zool 2011 61 739 750 10.3161/000345411X622561

[B62] Ugelvig LV Vila R Pierce NE Nash DR A phylogenetic revision of the *Glaucopsyche?* section (Lepidoptera: Lycaenidae), with special focus on the *Phengaris-Maculinea* clade Mol Phylogenet Evol 2011 61 237 243 10.1016/j.ympev.2011.05.016 21669295

[B63] Craft KJ Pauls SU Darrow K Miller SE Hebert PD Helgen LE Novotny V Weiblen GD Population genetics of ecological communities with DNA barcodes: an example from New Guinea Lepidoptera Proc Natl Acad Sci USA 2010 107 5041 5046 10.1073/pnas.0913084107 20202924 PMC2841870

[B64] Hulcr J Miller SE Setliff GP Darrow K Mueller ND Hebert PDN Weiblen GD DNA barcoding confirms polyphagy in a generalist moth, Homona mermerodes (Lepidoptera: Tortricidae) Mol Ecol Notes 2007 7 549 557 10.1111/j.1471-8286.2007.01786.x

[B65] Nice CC Gompert Z Forister ML Fordyce JA An unseen foe in arthropod conservation efforts: the case of *Wolbachia* infections in the Karner Blue butterfly Biol Conserv 2009 14 3137 3146

[B66] Muñoz AG Baxter SW Linares M Jiggins CD 2011 Deep mitochondrial divergence within a *Heliconius* butterfly species is not explained by cryptic speciation or endosymbiotic bacteria BMC Evol Biol 2011 11 358 10.1186/1471-2148-11-358 22151691 PMC3287262

[B67] Slatkin M Hudson RR Pairwise comparisons of mitochondrial DNA sequences in stable and exponentially growing populations Genetics 1991 129 555 562 1743491 10.1093/genetics/129.2.555 PMC1204643

[B68] Kodandaramaiah U Weingartner E Janz N Dalén L Nylin S Population structure in relation to host-plant ecology and *Wolbachia* infestation in the comma butterfly J Evol Biol 2011 24 2173 2185 10.1111/j.1420-9101.2011.02352.x 21745252

