# Supplementary material for: Contrasting genetic structure of rear edge and continuous range populations of a parasitic butterfly infected by Wolbachia
Source: BMC Evol Biol. 2013 Jan 18;13:14. doi: 10.1186/1471-2148-13-14 (PMC3558474; doi:10.1186/1471-2148-13-14)
Supplement: Additional file 1 — Table S1. Haplotype frequencies of the COI mtDNA gene in nine populations of M. arion from Italy and eleven populations from Poland. S2. Haplotype frequencies of the EF-1α nuclear gene in nine populations of M. arion from Italy and eleven populations from Poland. S3. Genetic differentiation (pairwise FST main text and supplementary table s3) at the COI gene (listed above the diagonal) and for EF-1α (listed below the diagonal) among nine populations of M. arion in Italy and eleven populations of M. arion in Poland (for full names of the locations see Tab. 1). Any significant FST main text and supplementary table s3 values are given in bold. [file 1471-2148-13-14-S1.doc]

**Table S1.** Haplotype frequencies of the COI mtDNA gene in nine populations of *M. arion* from Italy and eleven populations from Poland.

| Haplotype | Italy | | | | | | | | |  | Poland | | | | | | | | | | | Total |
| --- | --- | --- | --- | --- | --- | --- | --- | --- | --- | --- | --- | --- | --- | --- | --- | --- | --- | --- | --- | --- | --- | --- |
|  | CER | AUR | CET | BDR | LOA | CUN | VAL | CDF | VFE |  | GUG | SOW | PIA | TRU | HOR | ORC | SUK | HUT | BAB | KLU | SRO |  |
| 1 | - | 0.059 | - | 0.667 | 0.938 | 1.000 | 0.500 | 0.476 | 0.682 |  | 1.000 | 1.000 | 1.000 | 1.000 | 1.000 | 1.000 | 1.000 | 1.000 | 1.000 | 1.000 | 0.875 | **0.705** |
| 2 | - | - | - | - | 0.063 | - | - | - | - |  | - | - | - | - | - | - | - | - | - | - | - | **0.005** |
| 3 | - | 0.580 | 0.944 | 0.222 | - | - | - | - | - |  | - | - | - | - | - | - | - | - | - | - | - | **0.132** |
| 4 | - | - | 0.056 | - | - | - | - | - | - |  | - | - | - | - | - | - | - | - | - | - | - | **0.005** |
| 5 | 0.600 | 0.059 | - | - | - | - | 0.167 | 0.476 | 0.182 |  | - | - | - | - | - | - | - | - | - | - | - | **0.086** |
| 6 | 0.400 | 0.294 | - | - | - | - | - | - | - |  | - | - | - | - | - | - | - | - | - | - | - | **0.032** |
| 7 | - | - | - | - | - | - | - | - | 0.045 |  | - | - | - | - | - | - | - | - | - | - | - | **0.005** |
| 8 | - | - | - | - | - | - | - | - | 0.091 |  | - | - | - | - | - | - | - | - | - | - | - | **0.010** |
| 9 | - | - | - | - | - | - | - | 0.048 | - |  | - | - | - | - | - | - | - | - | - | - | - | **0.005** |
| 10 | - | - | - | 0.111 | - | - | - | - | - |  | - | - | - | - | - | - | - | - | - | - | - | **0.005** |
| 11 | - | - | - | - | - | - | 0.333 | - | - |  | - | - | - | - | - | - | - | - | - | - | - | **0.010** |
| 12 | - | - | - | - | - | - | - | - | - |  | - | - | - | - | - | - | - | - | - | - | 0.125 | **0.005** |

**Table S2.** Haplotype frequencies of the EF-1α nuclear gene in nine populations of *M. arion* from Italy and eleven populations from Poland.

| Allele | Italy | | | | | | | | |  | Poland | | | | | | | | | | | **Total** |
| --- | --- | --- | --- | --- | --- | --- | --- | --- | --- | --- | --- | --- | --- | --- | --- | --- | --- | --- | --- | --- | --- | --- |
|  | CER | AUR | CET | BDR | LOA | CUN | VAL | CDF | VFE |  | GUG | SOW | PIA | TRU | HOR | ORC | SUK | HUT | BAB | KLU | SRO |  |
| 1 | 0.700 | 0.775 | 0.952 | 0.750 | 0.441 | 0.460 | 0.375 | 0.643 | 0.729 |  | 0.250 | 0.800 | 0.550 | 0.500 | 0.312 | 0.150 | 0.500 | 0.250 | 0.300 | 0.400 | 0.375 | **0.588** |
| 2 | - | - | 0.024 | 0.035 | 0.206 | - | - | - | - |  | - | - | - | - | - | - | - | - | - | - | - | **0.017** |
| 3 | - | - | - | - | 0.235 | 0.420 | 0.375 | 0.119 | - |  | - | - | - | - | - | - | - | 0.083 | - | - | - | **0.075** |
| 4 | - | - | - | - | 0.030 | - | - | - | - |  | - | - | - | - | - | - | - | - | - | - | - | **0.020** |
| 5 | 0.300 | 0.075 | - | - | 0.059 | 0.120 | - | 0.095 | 0.042 |  | 0.679 | 0.200 | 0.450 | 0.429 | 0.562 | 0.600 | 0.318 | 0.458 | 0.600 | 0.550 | 0.438 | **0.243** |
| 6 | - | - | - | - | 0.030 | - | - | - | - |  | - | - | - | - | - | - | - | 0.042 | 0.100 | - | - | **0.008** |
| 7 | - | - | 0.024 | - | - | - | - | - | - |  | - | - | - | 0.071 | - | - | - | - | - | - | - | **0.004** |
| 8 | - | 0.075 | - | - | - | - | - | - | - |  | - | - | - | - | - | - | - | - | - | - | - | **0.006** |
| 9 | - | 0.025 | - | - | - | - | - | - | 0.146 |  | - | - | - | - | - | - | - | - | - | - | - | **0.015** |
| 10 | - | 0.025 | - | - | - | - | - | - | 0.083 |  | - | - | - | - | - | - | - | - | - | - | - | **0.010** |
| 11 | - | 0.025 | - | 0.107 | - | - | - | - | - |  | - | - | - | - | - | - | - | - | - | - | - | **0.008** |
| 12 | - | - | - | - | - | - | - | 0.024 | - |  | - | - | - | - | - | - | - | - | - | - | - | **0.002** |
| 13 | - | - | - | - | - | - | - | 0.024 | - |  | - | - | - | - | - | - | - | - | - | - | - | **0.002** |
| 14 | - | - | - | - | - | - | - | 0.048 | - |  | - | - | - | - | - | - | - | - | - | - | - | **0.004** |
| 15 | - | - | - | - | - | - | 0.125 | 0.048 | - |  | - | - | - | - | - | - | - | - | - | - | - | **0.006** |
| 16 | - | - | - | 0.071 | - | - | - | - | - |  | - | - | - | - | - | - | - | - | - | - | - | **0.004** |
| 17 | - | - | - | 0.036 | - | - | - | - | - |  | - | - | - | - | - | - | - | - | - | - | - | **0.002** |
| 18 | - | - | - | - | - | - | 0.125 | - | - |  | - | - | - | - | - | - | - | - | - | - | - | **0.002** |
| 19 | - | - | - | - | - | - | - | - | - |  | 0.071 | - | - | - | - | - | - | - | - | - | - | **0.004** |
| 20 | - | - | - | - | - | - | - | - | - |  | - | - | - | - | - | - | 0.091 | 0.042 | - | - | - | **0.006** |
| 21 | - | - | - | - | - | - | - | - | - |  | - | - | - | - | - | - | 0.046 | 0.042 | - | - | - | **0.004** |
| 22 | - | - | - | - | - | - | - | - | - |  | - | - | - | - | - | - | 0.046 | - | - | - | - | **0.002** |
| 23 | - | - | - | - | - | - | - | - | - |  | - | - | - | - | - | - | - | 0.042 | - | - | - | **0.002** |
| 24 | - | - | - | - | - | - | - | - | - |  | - | - | - | - | - | - | - | 0.042 | - | - | - | **0.002** |
| 25 | - | - | - | - | - | - | - | - | - |  | - | - | - | - | - | - | - | - | - | 0.050 | - | **0.002** |
| 26 | - | - | - | - | - | - | - | - | - |  | - | - | - | - | - | - | - | - | - | - | 0.188 | **0.006** |
| 27 | - | - | - | - | - | - | - | - | - |  | - | - | - | - | 0.125 | 0.050 | - | - | - | - | - | **0.006** |
| 28 | - | - | - | - | - | - | - | - | - |  | - | - | - | - | - | 0.100 | - | - | - | - | - | **0.004** |
| 29 | - | - | - | - | - | - | - | - | - |  | - | - | - | - | - | 0.050 | - | - | - | - | - | **0.002** |
| 30 | - | - | - | - | - | - | - | - | - |  | - | - | - | - | - | 0.050 | - | - | - | - | - | **0.002** |

**Table S3.** Genetic differentiation (pairwise *_ST_*) at the COI gene (listed above the diagonal) and for EF-1α (listed below the diagonal) among nine populations of *M. arion* in Italy and eleven populations of *M. arion* in Poland (for full names of the locations see Tab. 1)*.* Any significant *_ST_* values are given in bold.

|  | CER | AUR | CET | BDR | LOA | CUN | VAL | CDF | VFE | GUG | SOW | PIA | TRU | HOR | ORC | SUK | HUT | BAB | KLU | SRO |  |
| --- | --- | --- | --- | --- | --- | --- | --- | --- | --- | --- | --- | --- | --- | --- | --- | --- | --- | --- | --- | --- | --- |
| CER | - | **0.400** | **0.687** | **0.348** | **0.610** | **0.752** | **0.214** | 0.064 | **0.304** | **0.555** | 0.423 | **0.555** | 0.423 | 0.464 | 0.423 | **0.529** | **0.617** | **0.528** | **0.499** | **0.492** | CER |
| AUR | 0.056 | - | **0.121** | **0.313** | **0.567** | **0.672** | **0.437** | **0.514** | **0.428** | **0.547** | **0.487** | **0.547** | **0.487** | **0.505** | **0.487** | **0.534** | **0.581** | **0.535** | **0.520** | **0.512** | AUR |
| CET | **0.333** | **0.027** | - | **0.597** | **0.850** | **0.955** | **0.722** | **0.650** | **0.586** | **0.930** | **0.917** | **0.930** | **0.917** | **0.920** | **0.917** | **0.927** | **0.937** | **0.927** | **0.924** | **0.869** | CET |
| BDR | **0.139** | 0.026 | **0.043** | - | **0.083** | **0.194** | 0.055 | **0.311** | 0.056 | 0.050 | 0.000 | 0.050 | 0.000 | 0.000 | 0.000 | 0.034 | **0.089** | 0.034 | 0.016 | 0.031 | BDR |
| LOA | 0.060 | **0.178** | **0.241** | **0.181** | - | **0.026** | **0.193** | **0.420** | 0.088 | 0.000 | 0.000 | 0.000 | 0.000 | 0.000 | 0.000 | 0.000 | 0.000 | 0.000 | 0.000 | 0.000 | LOA |
| CUN | **0.198** | **0.345** | **0.421** | **0.357** | **0.059** | - | **0.377** | **0.504** | **0.133** | 0.000 | 0.000 | 0.000 | 0.000 | 0.000 | 0.000 | 0.000 | 0.000 | 0.000 | 0.000 | 0.156 | CUN |
| VAL | **0.260** | **0.466** | **0.499** | **0.467** | 0.042 | 0.000 | - | 0.158 | 0.000 | 0.160 | 0.043 | **0.160** | 0.043 | 0.080 | 0.043 | **0.137** | **0.219** | **0.137** | 0.111 | **0.109** | VAL |
| CDF | 0.000 | **0.045** | **0.101** | **0.080** | 0.047 | **0.167** | **0.187** | - | **0.163** | **0.379** | 0.319 | **0.379** | 0.319 | **0.338** | 0.319 | **0.367** | **0.412** | **0.367** | **0.353** | **0.358** | CDF |
| VFE | **0.094** | **0.026** | **0.070** | **0.063** | **0.200** | **0.362** | **0.441** | **0.070** | - | 0.049 | 0.000 | 0.049 | 0.000 | 0.010 | 0.000 | 0.039 | 0.073 | 0.039 | 0.026 | 0.046 | VFE |
| GUG | **0.253** | **0.475** | **0.678** | **0.531** | **0.221** | **0.243** | **0.377** | **0.256** | **0.465** | - | 0.000 | 0.000 | 0.000 | 0.000 | 0.000 | 0.000 | 0.000 | 0.000 | 0.000 | 0.016 | GUG |
| SOW | 0.000 | 0.017 | **0.168** | **0.089** | **0.112** | **0.267** | **0.394** | 0.004 | **0.067** | **0.392** | - | 0.000 | 0.000 | 0.000 | 0.000 | 0.000 | 0.000 | 0.000 | 0.000 | 0.000 | SOW |
| PIA | 0.000 | **0.208** | **0.459** | **0.288** | **0.105** | **0.206** | **0.315** | **0.058** | **0.228** | **0.122** | 0.087 | - | 0.000 | 0.000 | 0.000 | 0.000 | 0.000 | 0.000 | 0.000 | 0.016 | PIA |
| TRU | 0.000 | **0.175** | **0.428** | **0.249** | **0.084** | **0.189** | **0.259** | 0.037 | **0.197** | 0.126 | 0.057 | 0.000 | - | 0.000 | 0.000 | 0.000 | 0.000 | 0.000 | 0.000 | 0.000 | TRU |
| HOR | 0.047 | **0.297** | **0.541** | **0.358** | **0.120** | **0.191** | **0.263** | **0.112** | **0.303** | 0.041 | **0.184** | 0.000 | 0.000 | - | 0.000 | 0.000 | 0.000 | 0.000 | 0.000 | 0.000 | HOR |
| ORC | 0.112 | **0.357** | **0.552** | **0.405** | **0.160** | **0.204** | **0.263** | **0.178** | **0.368** | 0.006 | **0.246** | 0.047 | 0.042 | 0.000 | - | 0.000 | 0.000 | 0.000 | 0.000 | 0.000 | ORC |
| SUK | 0.000 | **0.147** | **0.303** | **0.202** | **0.096** | **0.203** | **0.260** | 0.046 | **0.177** | **0.130** | 0.039 | 0.000 | 0.000 | 0.007 | 0.061 | - | 0.000 | 0.000 | 0.000 | 0.000 | SUK |
| HUT | 0.079 | **0.312** | **0.452** | **0.343** | **0.088** | **0.097** | **0.121** | **0.132** | **0.330** | 0.022 | **0.192** | 0.043 | 0.031 | 0.010 | 0.011 | 0.044 | - | 0.000 | 0.000 | 0.054 | HUT |
| BAB | 0.132 | **0.381** | **0.568** | **0.422** | **0.114** | **0.124** | **0.176** | **0.166** | **0.385** | 0.009 | **0.268** | 0.066 | 0.060 | 0.015 | 0.005 | **0.070** | 0.000 | - | 0.000 | 0.000 | BAB |
| KLU | 0.083 | **0.337** | **0.579** | **0.403** | **0.139** | **0.201** | **0.304** | **0.138** | **0.340** | 0.011 | **0.228** | 0.000 | 0.000 | 0.000 | 0.000 | 0.020 | 0.004 | 0.003 | - | 0.000 | KLU |
| SRO | 0.100 | **0.335** | **0.502** | **0.362** | **0.153** | **0.213** | **0.223** | **0.176** | **0.344** | 0.088 | **0.222** | 0.081 | 0.042 | 0.047 | 0.051 | 0.056 | 0.021 | 0.028 | 0.051 | - | SRO |
|  | CER | AUR | CET | BDR | LOA | CUN | VAL | CDF | VFE | GUG | SOW | PIA | TRU | HOR | ORC | SUK | HUT | BAB | KLU | SRO |  |
